# Zinc Transporter ZIP14 Functions in Hepatic Zinc, Iron and Glucose Homeostasis during the Innate Immune Response (Endotoxemia)

**DOI:** 10.1371/journal.pone.0048679

**Published:** 2012-10-24

**Authors:** Tolunay Beker Aydemir, Shou-Mei Chang, Gregory J. Guthrie, Alyssa B. Maki, Moon-Suhn Ryu, Afife Karabiyik, Robert J. Cousins

**Affiliations:** 1 Food Science and Human Nutrition Department and Center for Nutritional Sciences, College of Agricultural and Life Sciences, University of Florida, Gainesville, Florida, United States of America; 2 Department of Biochemistry and Molecular Biology, College of Medicine, University of Florida, Gainesville, Florida, United States of America; College of Tropical Agriculture and Human Resources, University of Hawaii, United States of America

## Abstract

ZIP14 (slc39A14) is a zinc transporter induced in response to pro-inflammatory stimuli. ZIP14 induction accompanies the reduction in serum zinc (hypozincemia) of acute inflammation. ZIP14 can transport Zn^2+^ and non-transferrin-bound Fe^2+^ in vitro. Using a *Zip14^−/−^* mouse model we demonstrated that ZIP14 was essential for control of phosphatase PTP1B activity and phosphorylation of c-Met during liver regeneration. In the current studies, a global screening of ZIP transporter gene expression in response to LPS-induced endotoxemia was conducted. Following LPS, Zip14 was the most highly up-regulated Zip transcript in liver, but also in white adipose tissue and muscle. Using *ZIP14^−/−^* mice we show that ZIP14 contributes to zinc absorption from the gastrointestinal tract directly or indirectly as zinc absorption was decreased in the KOs. In contrast, *Zip14^−/−^* mice absorbed more iron. The *Zip14* KO mice did not exhibit hypozincemia following LPS, but do have hypoferremia. Livers of *Zip14−/−* mice had increased transcript abundance for hepcidin, divalent metal transporter-1, ferritin and transferrin receptor-1 and greater accumulation of iron. The *Zip14^−/−^* phenotype included greater body fat, hypoglycemia and higher insulin levels, as well as increased liver glucose and greater phosphorylation of the insulin receptor and increased GLUT2, SREBP-1c and FASN expression. The *Zip14* KO mice exhibited decreased circulating IL-6 with increased hepatic SOCS-3 following LPS, suggesting SOCS-3 inhibited insulin signaling which produced the hypoglycemia in this genotype. The results are consistent with ZIP14 ablation yielding abnormal labile zinc pools which lead to increased SOCS-3 production through G-coupled receptor activation and increased cAMP production as well as signaled by increased pSTAT3 via the IL-6 receptor, which inhibits IRS 1/2 phosphorylation. Our data show the role of ZIP14 in the hepatocyte is multi-functional since zinc and iron trafficking are altered in the *Zip14^−/−^* mice and their phenotype shows defects in glucose homeostasis.

## Introduction

Control of mammalian zinc homeostasis is maintained through zinc transporter activity. There are 24 zinc transporters that handle uptake, efflux and intracellular trafficking [1], [2]. Expression and function of some of these genes respond to a variety of physiological stimuli and/or dietary conditions, whereas others appear to be constitutively expressed. Some of these zinc transporters may exhibit loose selectivity and thus could contribute to the cellular distribution of other metals such as iron, manganese and cadmium [3]–[5]. This possibility would be influenced upon dietary intake levels of the normal metal substrate and environmental exposure of toxic metals or concurrent pathophysiological conditions. The evidence that these transporters participate in the transport of multiple cations is based on in vitro data and have not been tested in integrative systems.

Inflammation is initiated by pro-inflammatory cytokines that have profound effects of nutrient metabolism and utilization. During the acute phase response the liver prioritizes nutrient flows toward production of acute phase proteins and alters utilization of substrates for energy [6]. Trace elements are among those nutrients that exhibit atypical metabolic profiles during inflammation and infectious episodes [7], [8].

Using a global screening approach for the ZnT and Zip transporter genes, we identified that in the liver of mice treated with turpentine to create a sterile abscess, or lipopolysaccharide (LPS) to mimic initiation of innate immunity, *Zip14* was the gene most profoundly up-regulated by these pro-inflammatory conditions [9]. Furthermore, induction of *Zip14* and enhanced plasma membrane-associated ZIP14 was associated with increased zinc transport into hepatocytes via IL-6 and other mediators. Subsequently, we demonstrated that IL-1β and IL-1β-stimulated nitric oxide production increased transcriptional activity of *Zip14* and ZIP14 enhanced Zn^2+^ transport by hepatocytes [10]. IL-1β and nitric oxide induction are independent of IL-6. We hypothesized that these findings strongly suggest that ZIP14 is a component of the mediation of hypozincemia associated with acute inflammation. Subsequently, using a murine partial hepatectomy model, we identified that ZIP14 production is an important component of the liver regeneration process [11]. The latter is dependent on pro-inflammatory stimuli including TNF-α and IL-6 [12]. These recent findings on ZIP14 and regeneration amplify the scope of cellular processes that are influenced by this transporter. The mechanism was traced to control by Zn^2+^ of protein-tyrosine phosphatase 1B activity and c-Met phosphorylation.

Hypoferremia is also associated with inflammation and infection. Mechanisms that result in reduced serum iron in response to both acute and chronic stimuli focus on the regulatory peptide hepcidin. IL-6 is among the numerous factors that regulate hepcidin production in hepatocytes and leukocytes [13]. Hepcidin functions to control iron levels through regulation of the iron export transporter, ferroportin in enterocytes and macrophages, through a mechanism that regulates degradation of the transporter. The net effect is a reduction in the plasma iron concentration. Chronic hypoferremia is frequently called the anemia of inflammation [14].

While they are congruent events, the teleologic basis for the depression of plasma zinc and iron concentrations associated with acute infections and other pro-inflammatory conditions has not been firmly established. Hypozincemia has been suggested to support production of acute phase proteins, host defense proteins such as calprotectin [15], restriction of zinc from acquisition systems of pathogens [16], [17], some that influence virulens [18], immune cell function [19], and as shown in various systems, regulation of signaling pathways including inhibition of phosphatases [20]–[22], transcription factor binding activity and most likely ligand-receptor initiated events [23]. Limited data suggest that experimental prevention of the hypozincemia response is detrimental to the organism [24]–[26]. Similarly, hypoferremia has been suggested as a host defense process to restrict iron from pathogens [8,13 and 27]. Redistribution of iron to maintain energy metabolism in specific tissues is another likely reason for regulated tissue iron accumulation during inflammation and infection [8].

In the research presented here, a novel murine *Zip14^−/−^* model was used to demonstrate that ablation of the ZIP14 zinc transporter prevented the hypozincemia produced by LPS administration and that ZIP14 is a key component for controlling altered zinc homeostasis and signaling pathways in multiple tissues, including liver, white adipose tissue (WAT) and muscle, during endotoxemia. Moreover, *Zip14^−/−^* mice had a decreased capacity to absorb zinc. In contrast, the null mice exhibited increased iron absorption, but not hypoferremia in response to LPS. The *Zip14^−/−^* mice had a diminished IL-6 production after LPS, had more body fat, were hypoglycemic and exhibited characteristics of hyperinsulinemia.

## Materials and Methods

### Mice and Diets

*Zip14^+/−^* heterozygous mice of the C57BL/6 strain were obtained from the Mutant Mouse Research Resource Consortium at University of California-Davis via a contract. A breeding colony was established at the University of Florida to develop multiple generations to produce both homozygous (*Zip14^+/+^;* WT) and homozygous (*Zip14^−/−^*; KO) mice for use in these experiments. Genotyping was by PCR. Genomic characterization of the *Zip14^−/−^* strain has been presented previously [11]. KO and WT mice were used when 8–16 weeks of age. It is well documented that there is a greater iron content in female rodents compared to males [28]–[30]. Consequently, female mice were used for these experiments. In limited studies, CD-1 strain male mice (Charles River) were used when 8–12 weeks of age. For all of these studies all mice were maintained using standard rodent husbandry and received a commercial, irradiated diet (Harlan Teklad 7912) ad libitum and tap water.

**Figure 1:**
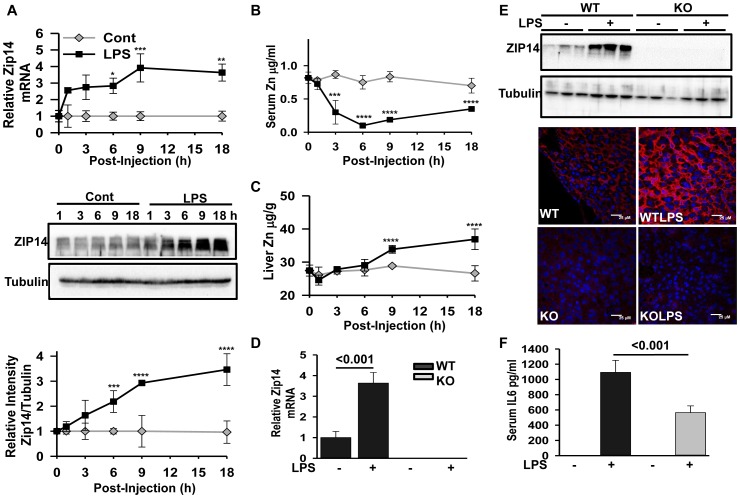
LPS differentially regulates ZIP14 expression in mice. Young adult mice received LPS (2 mg/kg, i.p.) or the same volume (0.5 mL) of saline (control), 1–18 hr before being killed. (A) Total RNA was isolated and *Zip14* mRNA was measured by qPCR and expressed relative to TBP mRNA as the normalizer. ZIP14 protein abundance was measured by western analysis of liver homogenates. Representative western blots from multiple mice (n = 3−4) were measured for ZIP14 abundance by densitometry. (B, C) Zinc concentrations in serum and liver, in µg/mL and µg/g respectively, were measured by AAS. (D, E) Comparison of Zip14 mRNA and ZIP14 protein in WT and *Zip14* KO mice 18 hr after LPS, as measured by qPCR and western analysis. Values are mean ± SD, n = 3−5. (E) ZIP14 protein is increased at the plasma membrane of hepatocytes of WT mice but not Zip14^−/−^ mice following LPS. Localization was by confocal microscopy using ZIP14 antibody and Alexa fluor594 secondary antibody and DAPI as the nuclear marker. (F) Serum IL-6 as measured by ELISA was used as an indicator of efficiency of LPS administration. The IL6 response from LPS was attenuated in the *Zip14^−/−^* mice. (* = P<0.05, ** = P<0.01, *** = P<0.001, **** = P<0.0001).

### Treatments

In some experiments, lipopolysaccharide (LPS) (*E. coli* serotype 055:B5; Sigma) was administered (2.0 mg/kg; i.p.) in phosphate buffered saline (PBS) or PBS alone for up to 18 hr before the mice were killed. In other experiments, ^65^Zn was provided by gavage (2 µCi/mouse in 250 µL of saline) to fasted mice 3 hr before being killed to assess zinc absorption and tissue distribution. In comparison experiments, ^59^Fe was provided by gavage (2 µCi/mouse in 250 µL of saline, 0.5 M ascorbic acid) to fasted mice. Mice were given food after 7 hr and were killed 24 hr later to assess iron absorption and tissue distribution. Specific activity of the ^65^Zn and ^59^Fe (Pekin Elmer) when used was 4.4 mCi/mg and 41.8 mCi/mg, respectively. Absorption and tissue accumulation of ^65^Zn and ^59^Fe was measured by gamma scintillation spectrometry. At the specified time for each isotope, the entire gastrointestinal tract was removed and the radioactivity in the carcass was measured [31]–[33]. The percent absorption of each radioisotope was calculated from those values and the dose administered then normalized to the body weight. In some experiments, radioactivity in serum and liver was measured. Mice were anesthetized by Isofluorane inhalation for injections, gavage and euthanasia by cardiac puncture. Protocols were approved by the University of Florida Institutional Animal Care and Use Committee.

**Figure 2:**
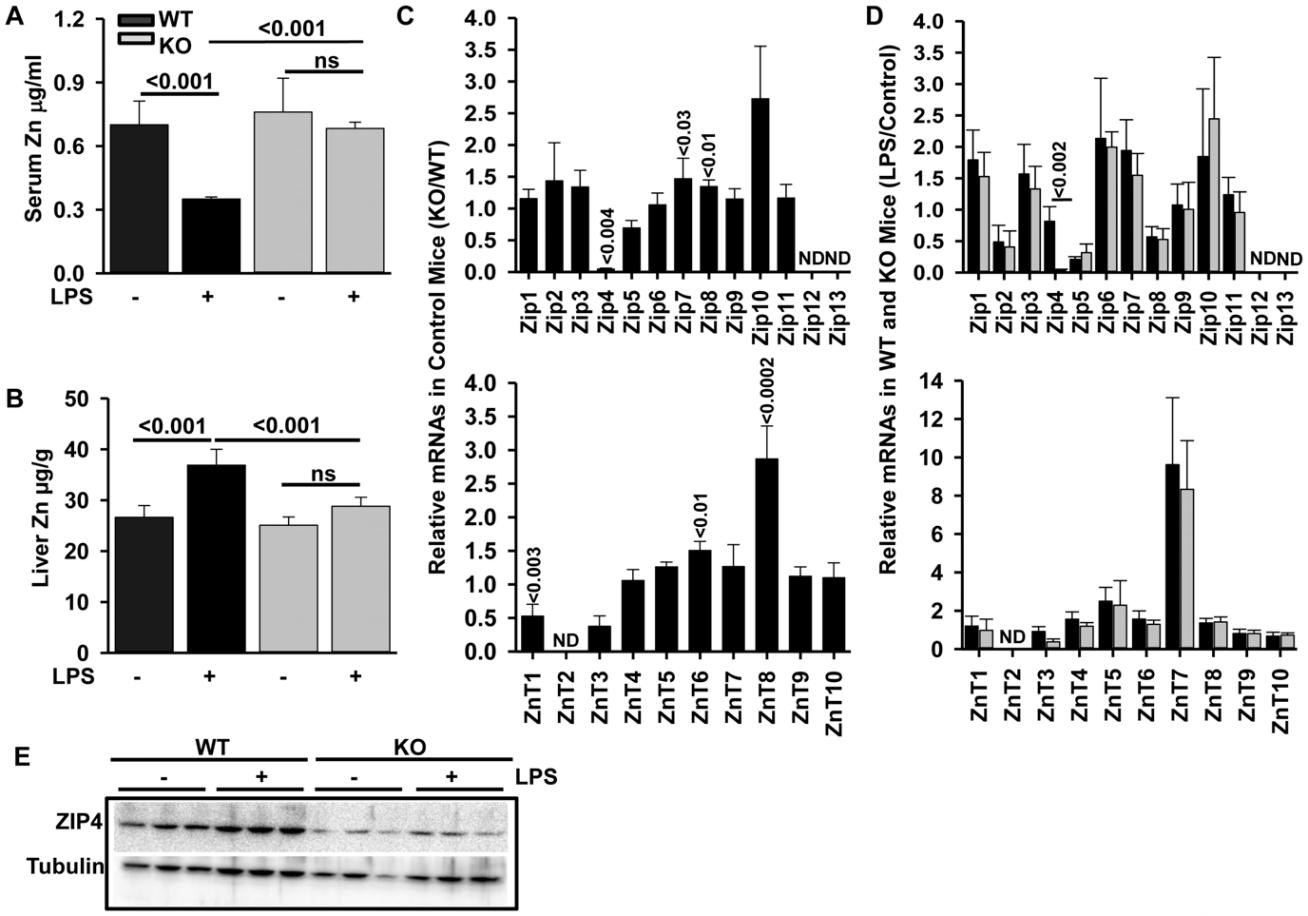
*Zip14* null mice do not have depressed serum zinc after LPS administration. WT and *Zip14^−/−^* mice were given LPS or saline administration (2 mg/kg; 0.5 mL; i.p.) 18 hr before being killed. (A, B) Zn concentrations in serum and liver were measured by AAS. (C, D) Global analysis of liver Zip and ZnT transcripts in KO and after LPS. Total RNA was isolated from the liver and *Zip14* mRNA was measured by qPCR and normalized to TBP mRNA. Values are mean ± SD, n = 3−5. (E) ZIP4 protein abundance in liver was shown by western analysis.

### Analytical Procedures

Blood was collected by cardiac puncture and serum was obtained by two-step centrifugation [34]. Zinc and iron concentrations of serum were measured by flame atomic absorption spectrophotometry (AAS) using serum diluted in MiliQ® water. To measure zinc and iron concentrations, weighed amounts of collected tissues were digested in HNO_3_ (90°C) for 3 hr prior to dilution in MilliQ® water and analysis by AAS [11]. Non-heme iron (NHI) concentrations in liver were determined colorimetrically (by ferrozine assay) [35]. Briefly, liver homogenates in water were diluted with (1∶1 ratio) protein precipitation solution (1 N HCl, 10% trichloroacetic acid) and incubated for 1 hr at 90°C. Clear supernatant was obtained by centrifugation at 16,000×g for 15 min. After 30 min incubation with the chromogen solution (0.508 mM ferrozine, 1.5 nM sodium acetate, 0.1% thioglycolic acid) absorbance was measured at 562 nm.

**Figure 3:**
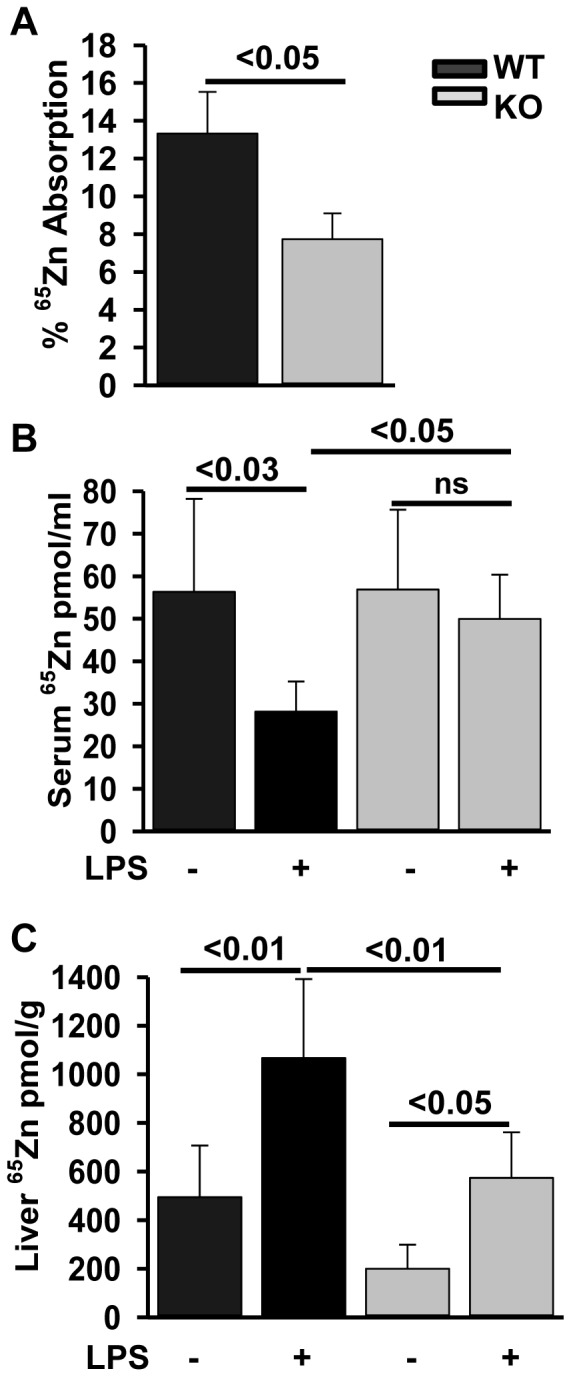
Zinc absorption and hepatic uptake is reduced in Zip14^−/−^ mice. Fasted mice received 2 µCi of ^65^Zn by gavage and were killed 3 hr later. (A) Percent absorption was calculated from the radioactivity administered. (B, C) Serum and liver uptake was calculated from the specific activity of the ^65^Zn. Values are mean ± SE, n = 5−12.

### RNA Isolation and qPCR

For isolation of RNA, tissues were placed in RNase Later (Ambion) and subsequently homogenized (Polytron) in TRI reagent (Ambion) [11], [21]. Total RNA was treated with Turbo DNA-free reagents (Ambion). Primer/probe sequences for the PCR reactions have been provided previously [9], [11] or TaqMan Gene Expression Assays for hepcidin, transferrin receptor-1/2 (TfR-1/2), divalent metal transporter 1 (DMT1), ferritin, glucose transporter 2 (GLUT2), phosphoenolpyruvate carboxykinase (PEPCK), sterol regulatory element-binding protein-1c (SREBP-1c), fatty acid synthase (FASN) and suppressor of cytokine signaling-3 (SOCS-3) were purchased from Applied Biosystems. One-step reverse transcriptase reactions (Applied Biosystems) were used for qPCR. TATA binding protein (TBP) mRNA was the normalizer for relative quantitation.

**Figure 4:**
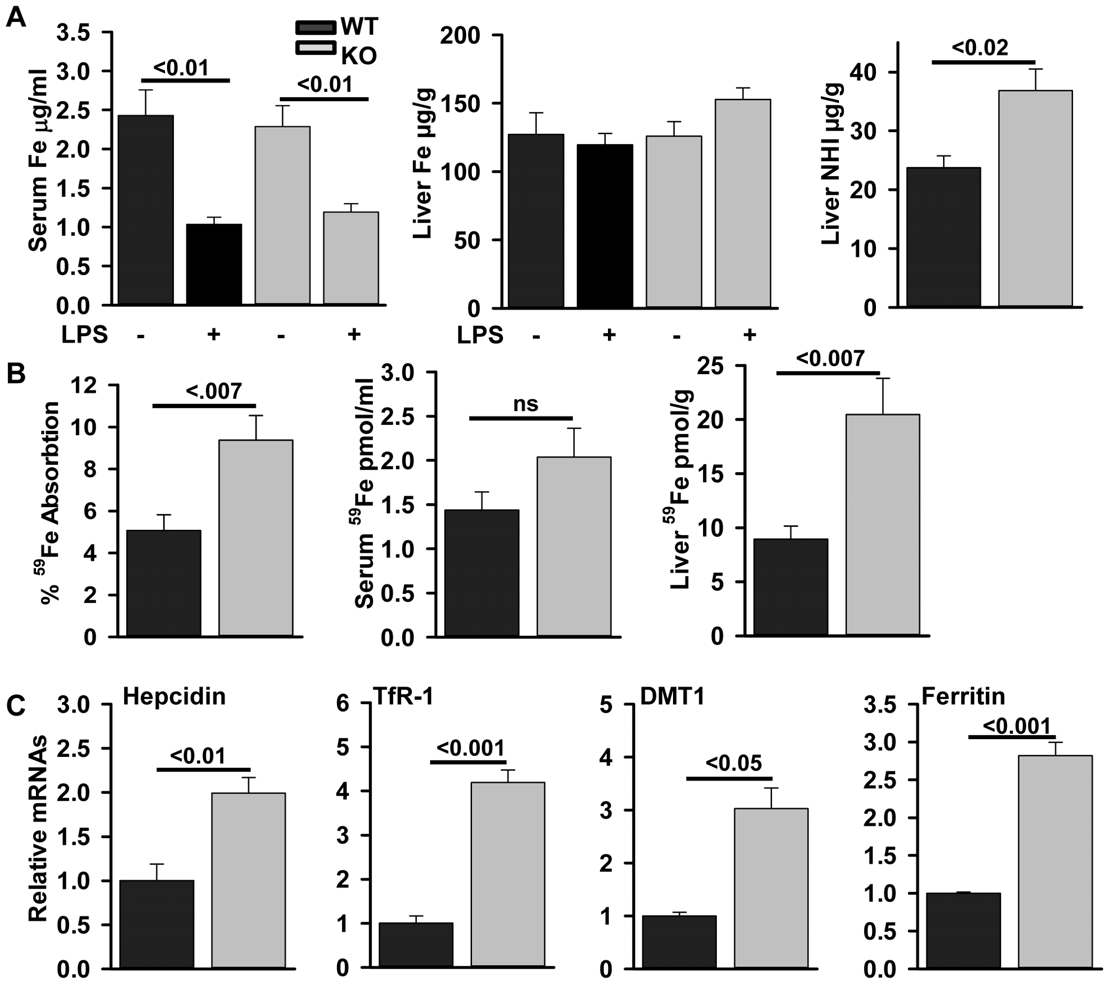
*Zip14^−/−^* mice exhibit normal iron absorption but altered iron homeostasis. (A) WT and Zip14^−/−^ mice were administered LPS (2 mg/kg or saline 0.5 mL; ip), 18 hr before being killed. (A) Serum and liver iron concentrations were measured by AAS. Liver non-heme-iron was measured colorimetrically. (B) Fasted WT and *Zip14^−/−^* mice received 2 µCi of ^59^Fe by gavage and were killed 24 hr later. Percent absorption was calculated from the radioactivity administered. Serum and liver iron uptake was calculated from the specific activity of the ^59^Fe. (C) Transcript abundance for liver hepcidin, TfR-1, DMT1 and ferritin was measured by qPCR and expressed relative to TBP mRNA as the normalizer. Values are mean ± SE, n = 5−10.

### Western Blotting and Immunohistochemistry

Western analysis used polyclonal rabbit antibodies against ZIP14, ZIP4 and ZnT8 produced in our laboratory and were affinity purified (Pierce) as previously described [9]. IR1β, phospho-IR1β (pY1146, pY1150/1151), P13K, phospho-P13K (pY488, pY199), IRS1/2, Akt, phospho-Akt (pS473), STAT3, phospho-STAT3 (pY705), IKB, phospho-lKB (pS32/36) antibodies were purchased from Cell Signaling Technology. Tissue samples were immediately flash frozen in liquid nitrogen. Tissues were homogenized (Potter-Elvehjem or Polytron) either in RIPA lysis buffer or non-denaturing lysis buffer (20 mM TrisHCl, 137 mM NaCl, 10% glycerol, 1% Triton X-100, 2 mM EDTA) containing protease and phosphatase inhibitors (SantaCruz, ThermoScientific). For immunoprecipitation, samples that were lysed in non-denaturing lysis buffer (1 mg/ml) were incubated overnight with sepharose conjugated pY antibody (Cell Signaling Technology). Proteins were separated by SDS-PAGE and transferred to nitrocellulose membranes. Immunoblots were visualized first by Ponceau Red staining and then with enhanced chemiluminescence to measure abundance by digital densitometry [11]. Tubulin (Abcam) was used as the loading control. Immunohistochemistry of liver and pancreas, used samples fixed with either 10% formalin in PBS or fresh frozen in an optimal cutting temperature compound. The ZIP14 and ZnT8 antibodies were followed with anti-rabbit IgG-Alexa Fluor 594 conjugate, while insulin antibody was followed with anti-goat IgG-Alexa Fluor 488 conjugate. Counterstaining of nuclei used 4, 6-diamond-no-2-phenylindole (DAPI). Visualization was by confocal microscopy. Light microscopy was used to detect pancreatic insulin and glucagon and liver red oil O staining.

**Figure 5:**
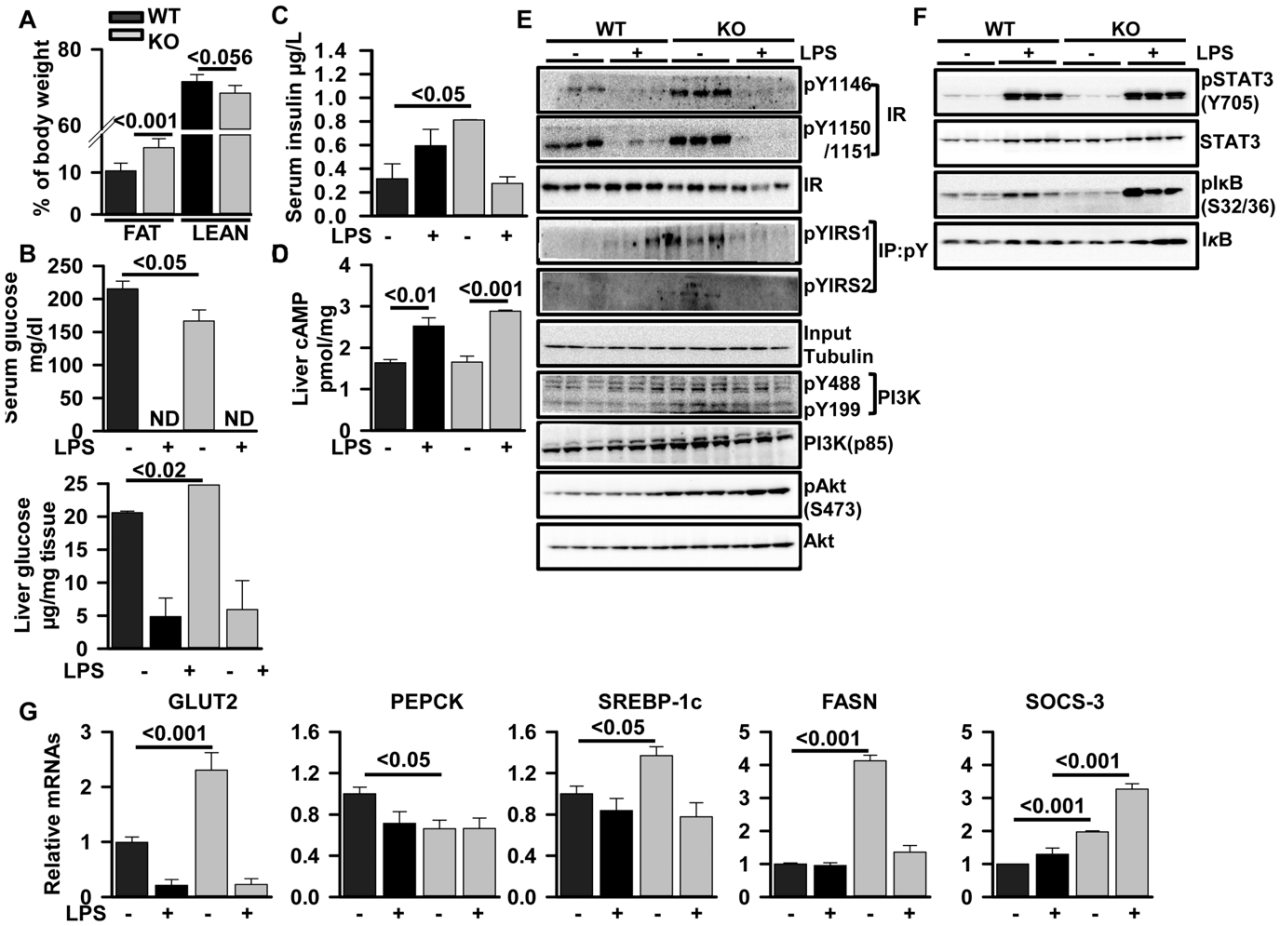
Deletion of *Zip14* in mice produces altered glucose homeostasis and IR functions. (A) Body composition of the WT and Zip14^−/−^ female mice was measured using a NMR Lean/Fat analyzer. (B) Serum and liver glucose from fed-mice were measured by OneTouch UltraMini and colorimetrically, respectively. (C, D) Serum insulin and liver cAMP were measured by ELISA. (E, F) Western analysis results from liver of three mice are shown for each treatment group. (G) Total RNA was isolated from livers and relative transcript abundance for GLUT2, PEPCK, SREBP-1c, FASN and SOCS-3 were measured by qPCR and expressed relative to TBP mRNA as the normalizer. Values are mean ± SE, n = 3−5.

### Other Methods

Serum IL-6 (BD Bioscience), insulin (Mercodia) and liver cyclic adenosine monophosphate (cAMP) (Arbor Assay) were measured by ELISA. Body composition was measured with Bruker Lean Fat Analyzer NMR [36]. Serum glucose concentration was measured by OneTouch Ultramini, while liver glucose was measured spectrophotometrically with glucose (GO) assay reagents (SIGMA), according to the manufacturer’s instructions. Proteins were precipitated as in the ferrozine assay and those supernatants were used for the assay.

**Figure 6:**
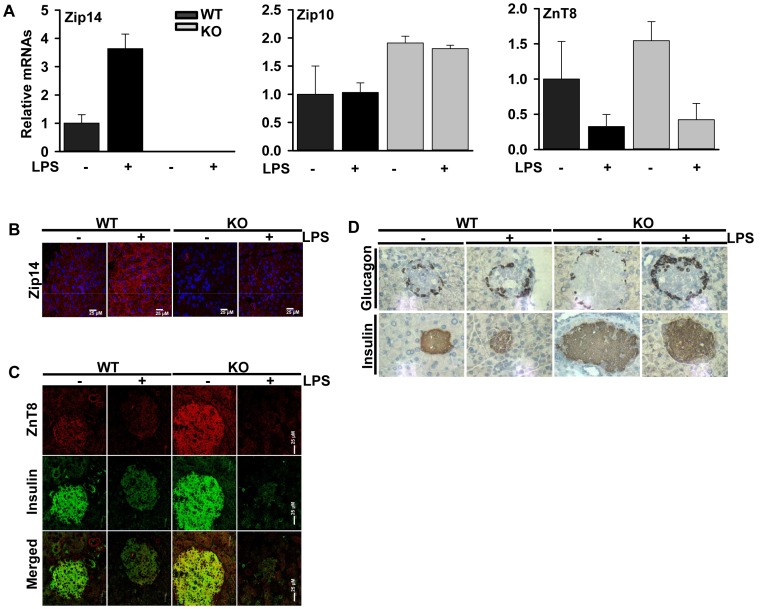
*Zip14^−/−^* mice exhibit altered zinc transporter expression and insulin and glucagon abundance in pancreas. (A) Total RNA isolated from pancreas and Zip14, Zip10 and ZnT8 mRNAs were measured by qPCR and TBP was used as a normalizer. Immunofluoresence microscopic analysis showing ZIP14 abundance (B) and ZnT8, insulin colocalization (C) in pancreas of the WT and *Zip14^−/−^* mice after LPS. (D) Immunohistochemical analysis of insulin and glucagon abundance in pancreatic islets of WT and *Zip14^−/−^* mice that were administered LPS (2 mg/kg or saline; 0. 5 mL; i.p.) 18 hr before being killed. Values are mean ± SD, n = 5.

### Statistical Analysis

Data are presented as the means ± SD or ± SEM. Significance was assessed by Student’s t-test or ANOVA. Statistical significance was set at p<0.05.

**Figure 7:**
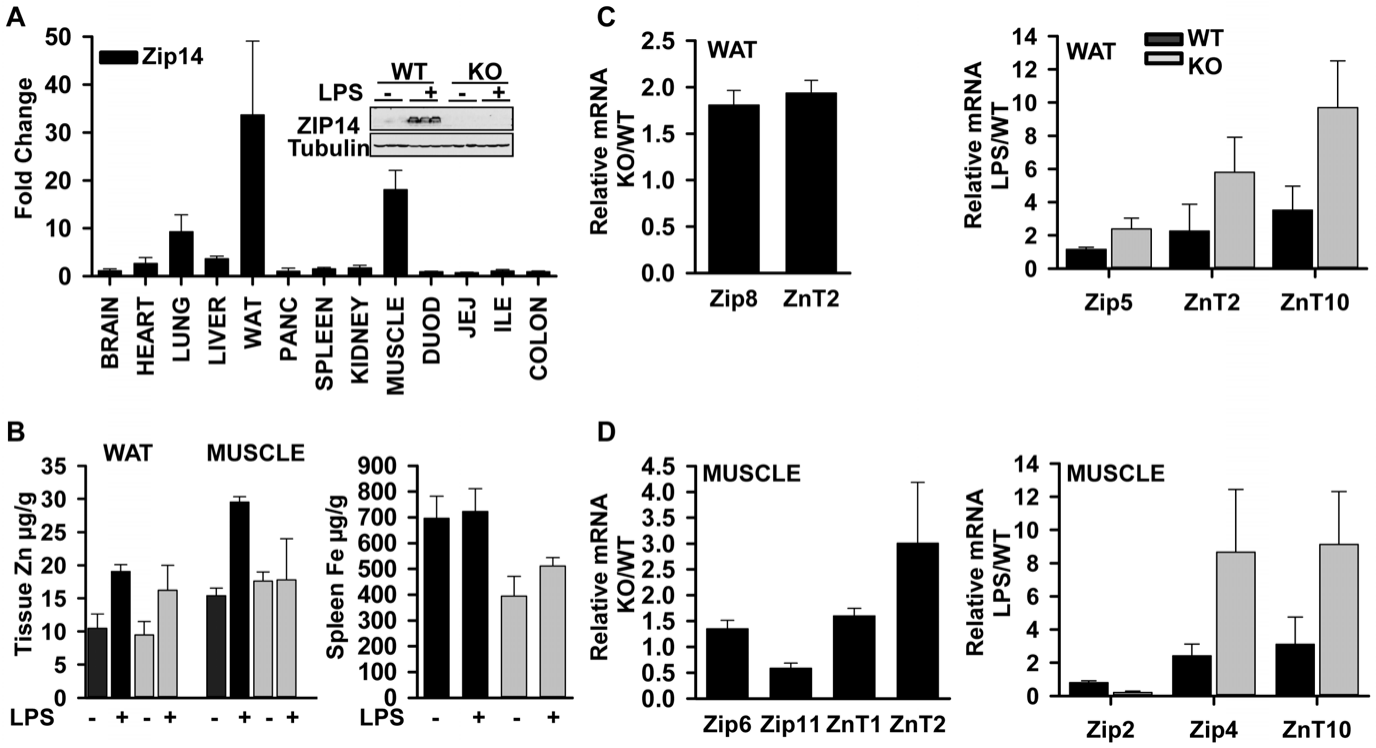
Zip14 expression is greatest in WAT and muscle following LPS and *Zip14* deletion produces atypical metal homeostasis. WT and *Zip14^−/−^* mice were administered LPS (2 mg/kg or saline; 0.5 mL; i.p.) 18 hr before being killed. (A) Total RNA was isolated from 13 tissues of the mice. Zip14 mRNA abundance was measured by qPCR and expressed relative to TBP mRNA as the normalizer. A western analysis shows ZIP14 expression in WAT. (B) Multiple tissues were removed from the mice and assayed for zinc and iron concentrations by AAS. Only tissues where there were significant differences in concentration are shown. (C, D) Global screening of ZnT and Zip mRNAs expressed in WAT and muscle was conducted by qPCR as described in (A). Only those ZnT and Zip transcripts that were significantly different in WT vs. *Zip14^−/−^* mice or following LPS treatment for WAT (C) and muscle (D) are shown. Values are mean ± SD, n = 3−5.

## Results

### ZIP14 is Up-regulated in Liver during Acute Phase Response

The LPS-induced proinflammatory response was used to examine hepatic ZIP14 expression. We conducted mRNA measurements showing the expression of *Zip14* mRNA in liver for up to 18 hr following LPS administration in female mice (Fig. 1A) of the C57BL/6 strain. The increase in hepatic ZIP14 protein abundance during the first 18 hr of the acute phase response is also shown (Fig. 1A). Hypozincemia occured rapidly after LPS administration (Fig. 1B). This is one of the sentinel signatures of the acute phase response. Of note is the significant increase in the liver zinc concentration at 9 and 18 hr following LPS administration (Fig. 1C). Therefore, subsequent experiments were conducted at 18 hr post LPS injection. To further explore the physiologic outcomes of ZIP14 expression we used *Zip14^−/−^* mice. The effectiveness of *Zip14* ablation is shown in that the KO strain show no measurable Zip14 mRNA (Fig. 1D) or ZIP14 protein in control and LPS-treated mice (Fig. 1E). Immunohistochemistry confirmed the increased ZIP14 abundance at the plasma membrane of hepatocytes following LPS treatment of WT mice and minimal signal in *Zip14* null mice (Fig. 1E). The serum concentrations of IL-6 were elevated in the LPS treated mice, but the response was attenuated (P<.001) in the null mice (Fig. 1F). Levels were undetectable in the saline treated mice, thus demonstrating effectiveness of LPS in initiating an inflammatory response. The *Zip14* null mutation did not produce liver damage as there was no detectable change in CD68, a marker of macrophage infiltration (Fig. S1). No markers of apoptosis were detected (data not shown).

**Figure 8:**
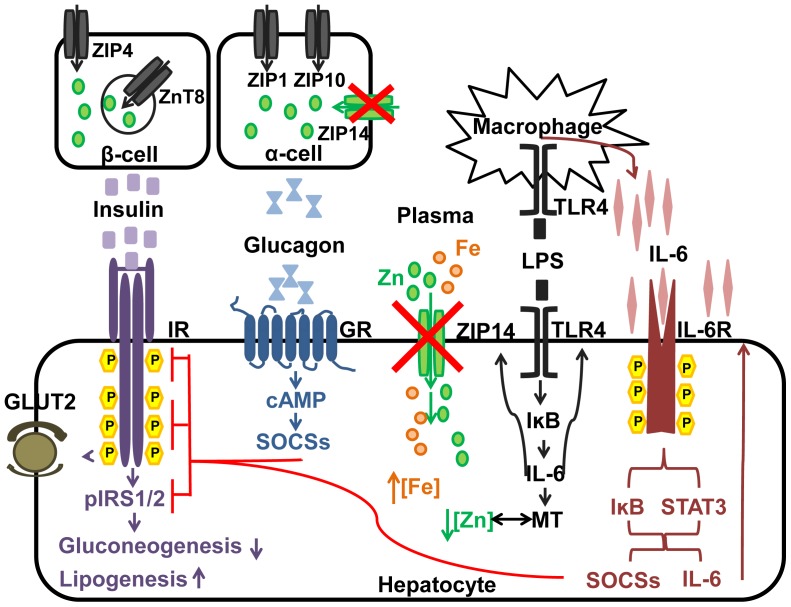
Model showing the influence of *Zip14* deletion in mice on hepatic zinc and iron metabolism and signaling pathways for glucose homeostasis during endotoxemia. The influence of the Zip14 null deletion on reduced liver zinc and increased iron uptake is shown. Up-regulation of Zip14 by LPS via toll like receptor 4 (TLR4) in liver and activation of the NFKB pathway leading to increased IL6, for an autocrine response, and from macrophage-produced IL-6. The suppression of IR activation by IL-6 and cAMP-induced SOCSs is also shown. The apparent reduction in glucagon production in pancreatic α cells and the effect on cellular cAMP in the Zip14 KO mice are proposed. We hypothesize that these signaling events produce hypoglycemia by a reduction in gluconeogenesis.

Administration of LPS resulted in significant (P<.001) hypozincemia in WT mice. This response was not observed in the *Zip14* null mice, however (Fig. 2A). Similarly, LPS produced an increase (P<.001) in liver zinc concentration in WT mice. LPS did not produce this response in *Zip14^−/−^* mice (Fig. 2B). Next we conducted a global analysis of ZnT and Zip transcript abundance in total liver RNA at 18 hr post-injection (Fig. 2C, D). The null mutation resulted in a number of changes, most notably a decrease in Zip4 (P<.004) to nearly undetectable levels and an increase in Zip10 (P<.007) mRNAs (Fig. 2C). Also notable was the increase in liver ZnT8 (P<.0002) in the KO mice (Fig. 2C). LPS increased Zip1, 3, 6, 7, and 10 mRNAs and ZnT7 mRNA equally in WT and *Zip14^−/−^* mice Fig. 2D). The depression in ZIP4 protein in the KO mice was confirmed by western analysis (Fig. 2E). Of note was that the induction of liver metallothionein (MT) mRNA by LPS, which was significantly attenuated in the *Zip14* KO mice (Fig. S2),since MT gene expression is proportional to intracellular zinc availability this response supports the notion that the induction of MT is the summation of LPS signaling and metal regulatory transcription factor 1 activation. Presumably newly transported zinc from ZIP14 activity, which raises the intracellular zinc concentration, hence the induction of MT was less in *Zip14* KO mice than WT mice following LPS (Fig. 2B).

### ZIP14 Influences Zinc and Iron Metabolism

The *Zip14^−/−^* mouse model allowed us to test the physiologic effects of ZIP14 function and the role played by ZIP14 in zinc metabolism in healthy mice and during LPS immune stimulation. Absorption of an oral dose of ^65^Zn was significantly less (P<.05) by about 50% in the *Zip14^−/−^* mice compared to WT mice (Fig. 3A). The amount of absorbed ^65^Zn found in the serum was significantly less (P<.03) in the LPS-treated WT mice (Fig. 3B). LPS did not influence ^65^Zn in serum of the KO mice, however. In contrast, hepatic incorporation of the orally administered ^65^Zn was increased (P<.01) in LPS-treated WT mice (Fig. 3C). The KO mice exhibited less hepatic ^65^Zn, but the magnitude of the response to LPS was retained. This latter finding was consistent with the decreased ^65^Zn absorption produced by *Zip14* ablation and the induction of another hepatic zinc transporter by LPS as demonstrated in Fig. 2C.

We measured tissue concentrations of iron and ^59^Fe absorption/metabolism to explore if the *Zip14* KO mice exhibited a phenotypic difference in iron handling based on in vitro evidence of iron transport mediated by ZIP14 (3, 5). Of major significance is that serum iron concentrations were reduced in both WT (P<.001) and KO (P<.001) mice upon LPS treatment (Fig. 4A). This indicates ablation of Zip14 expression did not prevent the hypoferremia of endotoxemia. Liver total iron content was not influenced by genotype or LPS. A significant increase (P<.02) in liver non-heme iron content in the *Zip14* null mice was detected, however (Fig. 4A).Orally administered ^59^Fe was used to access the influence of the *Zip14* null mutation on iron absorption and processing. ^59^Fe absorption during the 24 hr after oral administration was significantly (P<.007) increased in the ZIP14 KO mice (Fig. 4B). To further characterize the metabolic consequences of the *Zip14* null mutation on iron metabolism, uptake of ^59^Fe into serum and liver was evaluated. The ^59^Fe content of the serum was not influenced by the *Zip14* deletion (Fig. 4B). There was a significant increase (P<.04) in liver ^59^Fe in the null mice, however (Fig. 4B). A number of iron-responsive parameters were also examined. LPS increased hepcidin mRNA in the WT mice (data not shown). Similarly, control (saline-treated) *Zip14* null mice had a doubling of hepcidin transcript abundance (P<.01) compared to the WT mice (Fig. 4C). TfR-1 mRNA was significantly (P<.001) up-regulated in the liver of *Zip14^−/−^* mice (Fig. 4C). Of particular note is the increased DMT1 (P<.05) mRNA and ferritin (P<.001) mRNA also found in the liver of the *Zip14* null mice. However, TfR-2 mRNA levels were not altered (data not shown). These data collectively suggest a dysfunctional signaling for iron homeostasis in *Zip14^−/−^* mice. Specifically this is a pattern that leads to enhanced hepatic iron uptake via DMT1 and TfR-1 with deposition as ferritin.

### Hepatic Insulin and IL-6 Pathways are Differentially Regulated in *Zip14^−/−^* Mice

While there are clearly dysfunctional aspects to the metabolic pathways for zinc and iron, the *Zip14* null mice also showed some gross phenotypic characteristics that were readily apparent. For example, the null mice appeared to have difficulty in standing to consume food in cages where the food was placed in suspended feeders. The null mice, especially females, appeared to be smaller than the WT counterparts. Some of these characteristics were recently reported by others using *Zip14^−/−^* of different origin than those used in our current studies [37]. Using a Lean/Fat Analyzer, we found the *Zip14* null mice had 5% more body fat (Fig. 5A). This finding led us to compare blood chemistry parameters in the WT vs. KO mice. Salient among these parameters of fed-mice was a significant reduction (P<.05) in serum glucose (Fig. 5B) and a two-fold greater (P<.05) serum insulin concentration in the null mice (Fig. 5C). Also of note was the severe hypoglycemia in both genotypes produced by LPS treatment (Fig. 5B). The glucose content of the liver was increased in the *Zip14* null mice (Fig. 5B). Reduced hepatic glucose stores were observed upon LPS administration. Liver cAMP levels were up-regulated by LPS injection, however they were not altered by genotype (Fig. 5D). There was a significant increase (P<.001) in GLUT2 mRNA and a significant (P<.05) reduction in gluconeogenic enzyme PEPCK mRNA in *Zip14* null mice (Fig. 5G). LPS reduced both hepatic glucose and GLUT2 mRNA equally in both WT and *Zip14* null mice. Of note is that expression of both the lipogenic SREBP- 1c and FASN transcripts were significantly up-regulated (P<.05 and P<.001 respectively) in the livers of *Zip14* null mice (Fig. 5G). These increases were eliminated with LPS treatment. An increase in hepatic lipid content was shown by increased Red Oil O staining in the null mice (Fig. S3). The *Zip14* null mutation had a striking influence on hepatic insulin receptor (IR) phosphorylation. Phosphorylation of pY1146, pY1150 and pY1151 sites of IR and tyrosine phosphorylation of IRS1 and IRS2 was markedly increased (Fig. 5E). In addition, downstream targets of IR, PI3K and Akt also exhibited increased phosphorylation in the livers of the null mice (Fig. 5E).

As shown earlier *Zip14* is regulated by IL-6 [9]. Therefore, we investigated the influence of the *Zip14* null mutation on IL-6 pathways. The LPS-stimulated increase in serum IL-6 was attenuated with *Zip14* null mice (Fig. 1F). IL-6 synthesis after LPS stimulation is regulated by STAT3 and 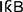 phosphorylation. After LPS administration, while pSTAT3 was increased equally in both genotypes, p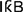 was increased to a far greater extent in the *Zip14* null mice (Fig. 5F). Expression of the mRNA for suppressor of cytokine signaling-3 (SOCS-3), a downstream target gene of the STAT3- 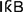 pathway, was markedly increased in the LPS-injected null mice (P<.001) (Fig. 5G). Of note is that the SOCS proteins are negative regulators of both IL-6 and insulin signaling [38].

### Pancreatic Insulin and Glucagon Secretion is Influenced by *Zip14^−/−^*

The influence of the null mutation and LPS administration on Zip14, Zip10 and ZnT8mRNA are shown in Fig. 6A. Immunofluorescent micrographs of ZIP14 and ZnT8 protein are presented in Fig. 6 B and C, respectively. Recent reports have implicated specific zinc transporters in pancreatic endocrine functions [39], [40] with Zip10, Zip14, and ZnT8 being those with high abundance. Of note is that both *Zip10* and *ZnT8* were up-regulated in the pancreas of the *Zip14* null mice (Fig. 6A). Furthermore, colocalization of ZnT8 with insulin was shown in pancreatic tissue (Fig. 6B). ZnT8 did not reach the statistical significance at the mRNA level, however immunofluorescence results showed clear up-regulation of ZnT8 in pancreatic islets of ZIP14 KO mice. This could be explained by the fact that mRNA measurements were conducted from RNA extracted from whole pancreas not specifically pancreatic islets.

As the pancreas is the site of insulin and glucagon production, we next investigated expression of these hormones in the pancreas of WT and *Zip14* null mice using immunohistochemistry (Fig. 6D). Insulin content of Beta cells was greater in the *Zip14* KO mice with characteristic hyperplasia of these cells (Fig. 6C, D). In addition, glucagon abundance was increased by LPS, but to a far greater extent in the *Zip14* null mice Fig. 6D).

### ZIP14 is Up-regulated in Multiple Tissues during Acute Phase Response

The signaling pathways identified in the liver suggest ZIP14-mediated zinc transport and signaling extends to other organ systems. For this evaluation, we determined the tissue specificity of *Zip14* mRNA expression following LPS administration in multiple tissues. Of the 13 tissues examined, the WAT and muscle had the highest Zip14 expression following LPS and were much higher than that of the lung, liver, and heart (Fig. 7A). For comparison, *Zip14* mRNA abundance in CD-1 male mice was also measured in tissues after LPS (Fig. S4A). Most notable was far greater expression in WAT and muscle comparable to what was observed in the C57BL/6 strain (Fig. 7A). LPS did not appreciably influence *Zip14* mRNA levels in the intestinal tract of either strain (data not shown). Of potential significance is that in muscle, LPS did not induce the *Zip14a* transcript, but did induce the *Zip14b* transcript (Fig. S 4B and C). We measured zinc and iron concentrations in liver, WAT, muscle, lung, pancreas and spleen. LPS significantly increased the zinc concentrations in liver (Fig. 2B), WAT and muscle of WT mice (Fig. 7B). This increase was not observed in liver and muscle of the *Zip14* null mice. In contrast, in WAT of the null mice, LPS elevated the zinc concentration as in WT mice. This suggests that in WAT another transporter is used to provide the additional zinc during an inflammatory response. The zinc concentration of the lung, pancreas and spleen were not altered by genotype or LPS (data not shown). The only significant change in total tissue iron of the six tissues examined was the reduced iron concentrations found in the spleens of the *Zip14* null mice (Fig. 7B). A global screen of all *ZnT* and *Zip* transcripts in WAT and muscle revealed remarkable selectivity with respect to expression differences produced by the *Zip14* null mutation and the immune response to LPS. Transcript abundances that were significantly changed by genotype or LPS administration (P<.05) in WAT and muscle are shown in Fig. 7C and D, respectively. Most notable was the influence of the *Zip14* null mutation on the up-regulation of ZnT2 and Zip8 and major increases in ZnT2 and ZnT10 after LPS in WAT (Fig. 7C). Similarly, the increases in ZnT2, Zip4 and ZnT10 mRNA abundance as influenced by genotype and LPS in muscle were striking (Fig. 7D).

## Discussion

The experiments presented in this report demonstrate that the *Zip14* null mutation in mice produces both altered zinc metabolism, altered segments of iron metabolism and altered signaling functions that influence glucose homeostasis. In addition, many of these processes are influenced by LPS and many of those are altered in the *Zip14* null mice. These findings suggest that the biological role of ZIP14 extends beyond metal ion trafficking and is not limited to the liver,specifically, the unexpected finding of the marked influence of *Zip14* deletion on glucose homeostasis.

Cells have elegant systems to control the inward and outward transport of zinc, iron and other metal ions [1], [2], [8], [41]. For a given cell type, uptake mechanisms are influenced by enteric absorption of these metals from the diet and physiologic controls for their utilization. These mechanisms provide a strong control over the levels of these ions in the peripheral circulation. For decades endotoxin has been known to have influences on zinc and iron metabolism, particularly producing hypozincemia and hypoferremia [9,42 and 43]. Therefore, LPS-induced endotoxemia was chosen as a model to evaluate ZIP14 function in an integrative null deletion model.

As shown in this report, after an oral dose of ^65^Zn the lack of ZIP14 resulted in a substantial reduction in zinc absorption from the intestinal tract. In contrast, the serum zinc concentration is maintained at normal levels in the *Zip14* null mice. This demonstrates that another transporter(s) is sufficient to sustain these levels. In that context, it is clear that the zinc transporter ZIP4 is the major determinant of dietary zinc absorption [44], [45] and is a major factor in supplying sufficient zinc to meet the dietary requirement [7]. The human zinc malabsorption syndrome Acrodermatitis Enteropathica is produced by mutations in Zip4 producing dermatologic and immunologic defects [46], [47]. Since this condition can be prevented by supplemental zinc [47], other transporters expressed in the gastrointestinal-tract, e.g. ZIP14, must contribute to enteric zinc absorption and cellular uptake. Hepatic Zip4 mRNA is reduced to nearly undetectable levels with a corresponding increase in Zip10 mRNA in the *Zip14* null mice. These results are interesting since both genes tend to up-regulate upon dietary zinc restriction and are under various control mechanisms [44,48 and 49]. It is of considerable interest that neither Zip14 nor Zip10 mRNA levels in the intestine are influenced by the *Zip14* null mutation. We interpret these findings to be a reflection of dysfunctional zinc homeostasis with *Zip14* ablation. Further experiments are needed to define how the differing transport capabilities of these proteins maintain hepatic zinc concentrations (Fig. 2A). Of particular interest is that ZIP14 appeared to influence iron absorption under the conditions used in these experiments. Assayed 24 hr after oral gavage; ^59^Fe absorption was not different between WT and *Zip14^−/−^* mice. Considering the differences in absorption kinetics for zinc and iron in rodents, the selection of 3 hr and 24 hr post-gavage are realistic time points to gain initial estimates of these rates [31]–[33].

We have shown with transfection experiments using HEK293T cells and, in collaboration with others using Xenopus oocytes, that ZIP14 is capable of transporting both zinc and iron [3], [5]. Similarly, using in vitro model systems, others have also shown manganese is another substrate that can be transported by ZIP14 [4]. Hence, there is the need to compare these in vitro findings with what is observed with an integrative model, since the available plasma/cellular concentrations of these metal ions in vivo are markedly different from metal ion concentrations available under in vitro conditions.

The hepatic metabolism of both zinc and iron in response to LPS has been extensively studied. LPS-induced changes in zinc metabolism initially focused at the mechanistic level on MT [42,46,47,50 and 51]. This protein was proposed as the recipient of zinc during the hypzoncemia-related hepatic zinc accumulation during acute endotoxemia. Liquid chromatography experiments with co-migration of zinc and MT led to that conclusion. Subsequently, MT has been viewed as a zinc buffer where the β cluster of the protein provides physiologic metal exchange, but α cluster having a greater binding affinity for some metals, serves a detoxification function [52]. For over two decades evidence has been accumulating that the zinc fluxes created by these metabolic events are primarily functioning for the cell signaling role of zinc rather than the catalytic or structural roles for this nutritionally essential metal [22], [23]. The role of zinc in cell signaling in vivo involves inhibition of phosphatases and other enzymes that influence immune responses and cell proliferation [11], [21]. In this respect, the in vitro effects of zinc, demonstrated over many years, are merging with information through effects produced in specific cell types generated by differential zinc transporter expression. We have stressed that the various modes of ZnT/Zip gene regulation, and marked differences in expression among cell types, point to specific effects on signaling [1], [2]. The high expression of Zip10 in brain, ZnT8 in pancreatic β cells, ZnT2 in secretory cells and Zip8 in T-lymphocytes are but a few examples.

It has been established that DMT1 is an iron transporter that is important for enteric iron absorption, NTBI delivery into cells at the plasma membrane and iron transport to the cytoplasm from an endosomal localization [8]. The latter delivers iron to mitochondria and the intracellular iron pool which includes ferritin. As with zinc, hepatic iron metabolism is regulated by innate immune responses and infectious stimuli. When modeled using LPS there is acute hypoferremia and body iron redistribution produced by hepcidin, an iron regulatory peptide synthesized in the liver in response to pro-inflammatory conditions or excess serum iron [41]. It has been proposed that hepcidin is responsible for orchestrating rapid changes in iron metabolism in response to pro-inflammatory stimuli [43]. We did not observe major changes in serum iron but NHI was increased in the *Zip14^−/−^* genotype compared to WT mice. Furthermore, the response to LPS produced comparable hypoferremia in both genotypes. This suggests ZIP14 does not function in either the hypoferremia or the liver iron accumulation associated with LPS administration. In contrast, absorption of ^59^Fe from the gastrointestinal tract and uptake the liver was significantly increased in the ZIP14 KO mice. Significant increases were noted in the *Zip14* null mice for hepatic hepcidin, TfR-1, DMT1 and ferritin mRNA expression as well as increased non-heme iron in liver. The increases in hepcidin and ferritin mRNA expression, plus the increased uptake of newly acquired hepatic iron (^59^Fe) in the *Zip14^−/−^* mice are consistent with a response to an iron overload situation. This could be driven by increased DMT1 activity and/or increased hepatic uptake of Tf-bound iron. Hepatocytes do not require functional DMT1 for iron uptake, however [53]. These in vivo data argue against a role for ZIP14 in cellular iron uptake, as shown with in vitro experiments but could reflect a block in an iron export pathway from an intracellular iron pool when ZIP14 is not present. The increased expression of TfR-1 and ferritin are in agreement with data on iron accumulation in zinc deficient 3T3 cells in culture [54]. Hence the hepatocytes from the *Zip14^−/−^* mice might be responding to a cellular zinc deficiency. Further experiments will address this point.

Increased body fat and hypoglycemia of the *Zip14^−/−^* mice were not expected findings. Increases in GLUT2, SREBP-1c and FASN mRNAs in the null mice are consistent with enhanced lipogensis and glucose utilization. Clearly enhanced phosphorylation of the IR was found in the *Zip14^−/−^* mice. Similarly, enhanced phosphorylations of PI3K and Akt, as found in the Zip14−/− mice, are indicators of enhanced glucose transport and lipogenesis and inhibition of lipolysis. Up-regulation of SOCS-3 in the null mice is compatible with the NFkB regulation and inhibition of insulin signaling [38]. Assuming that the high levels of the hepatic glucose levels are of endocrine origin, we focused on specific parameters of the pancreas of the *Zip14^−/−^* mice. The LPS-induced five-fold increase in *Zip14* mRNA and protein in the intact pancreas suggests that the loss of ZIP14 in the null mice is physiologically relevant. Considerable indirect evidence has linked zinc to glucose homeostasis [55]. Zinc deficient rats tend to have a diabetic phenotype [56]. Acute administration of zinc in vivo produces a transient hyperglycemic effect [57]. In vitro addition of zinc to rodent hepatocytes has been shown to stimulate glycolysis. This effect is believed to be produced by increased intracellular zinc levels [58], [59]. MT has been implicated in the stimulation of glycolysis by zinc as the effect is markedly diminished in hepatocytes from MT1-2^−/−^ mice [59]. Of note is that zinc accumulation and MT synthesis are stimulated by glucagon [60] and the inhibitory influence of glucagon on glycolysis is reversed by zinc [58]. While only measured at the transcript level, Zip14 expression was relatively high in a glucagon-producing cell line (x-TCG) and pancreatic islets from mice [40]. Consequently, the influences of zinc on glucagon secretion could be altered with *Zip14* ablation or by LPS via stimulation of ZIP14 transport activity. Glucagon secretion from α-cells occurs during hypoglycemia. Recent studies of this secretory process during glucose deprivation suggests regulation by zinc which acts as a switch to open K^+^ATP channels in α cells [61]. Furthermore, stimulation of MT synthesis may sufficiently reduce labile zinc in hepatocytes with subsequent stimulation of glycolysis. A key gluconeogenic enzyme, fructose 1, 6-bisphosphatase, is inhibited by zinc [62] which also influences activity of this enzyme in vivo [63]. The abnormal glucose homeostasis observed in the *Zip14^−/−^* mice could also result from the altered iron trafficking observed with this null mutation. Excess iron uptake has specifically been related to TfRs redistribution from an intracellular membrane compartment to cell surface caused by insulin [64]. Furthermore, TfRs have been shown to colocalize with insulin-responsive glucose transporters [64].

The demonstration of increased Zip14 mRNA in WAT and muscle following LPS, as reported here, places these findings within the context of being a reflection of an increased demand for zinc during the pro-inflammatory state. Numerous studies have suggested that the hepatic zinc accumulation concurrent with the acute phase response is necessary for enhanced protein synthesis or energy production [9], [51]. As a major site of IL-6 production [65], muscle is a participant in innate immunity. IL-6 synthesis in muscle occurs via a NF-kB requiring transcriptional process [66]. Hence this mode of regulation is in agreement with the enhanced IkB expression found in the *Zip14^−/−^* mice. In adipocytes, LPS signals an ERK1/2 pathway to stimulate lipolysis [67] presumably to meet the demands of enhanced energy expenditure during inflammation. A perspective on Zip14 expression in WAT should include recognition that the *Zip14* gene was first identified in differentiating adipocytes [68]. Furthermore, adipocytes are secretory cells [69], releasing a variety of factors including cytokines which are produced during inflammation associated with metabolic diseases. Of note is that zinc may be required for leptin secretion and differential expression of both ZnT and Zip genes in adipose tissue from lean vs. obese subjects [70]. Zip14 expression was not included in that analysis. In addition, lipid metabolism has now been linked to the innate immune response [71]. Clearly future emphasis will need to be placed on ZIP14-related functions in WAT and muscle within the context of inflammation and energy expenditure. We present a model that incorporates our analysis of how LPS administration to *Zip14* null mice influences intracellular signaling (Fig. 8). It includes the LPS induced zinc accumulation and enhanced IL-6 production/secretion. The inhibitory influence on the IR, via SOCS-3, as induced by IL-6, supports the influence of ZIP14 on glycolysis in liver. The net effect of the null mutation is an influence on insulin utilization.

The research presented here provides evidence that ZIP14 is a functional component of the hepatic response to acute inflammation that influences both utilization of metal ions and energy metabolism. Furthermore, the data provides the first link of the ZIP14 zinc transporter with signaling processes in tissues of high metabolic activity.

## Supporting Information

Figure S1 **The Zip14 null mutation did not produce liver damage as there was no detectable change in CD68, a marker of macrophage infiltration.**
(TIF)

Figure S2 **Comparison of MT mRNA WT and Zip14 KO mice 18 hr after LPS, as measured by qPCR.**
(TIF)

Figure S3 **The increase in hepatic lipid content was shown by increased Red Oil O staining in the null mice.**
(TIF)

Figure S4 **Zip14 mRNA abundance in CD-1 male mice and was measured in tissues after LPS. Zip14a and Zip14b transcripts were measured in liver, WAT and muscle.**
(TIF)
