# Supplementary figures and images for: Zinc Transporter ZIP14 Functions in Hepatic Zinc, Iron and Glucose Homeostasis during the Innate Immune Response (Endotoxemia)
Source: PLoS One. 2012 Oct 24;7(10):e48679. doi: 10.1371/journal.pone.0048679 (PMC3480510; doi:10.1371/journal.pone.0048679)

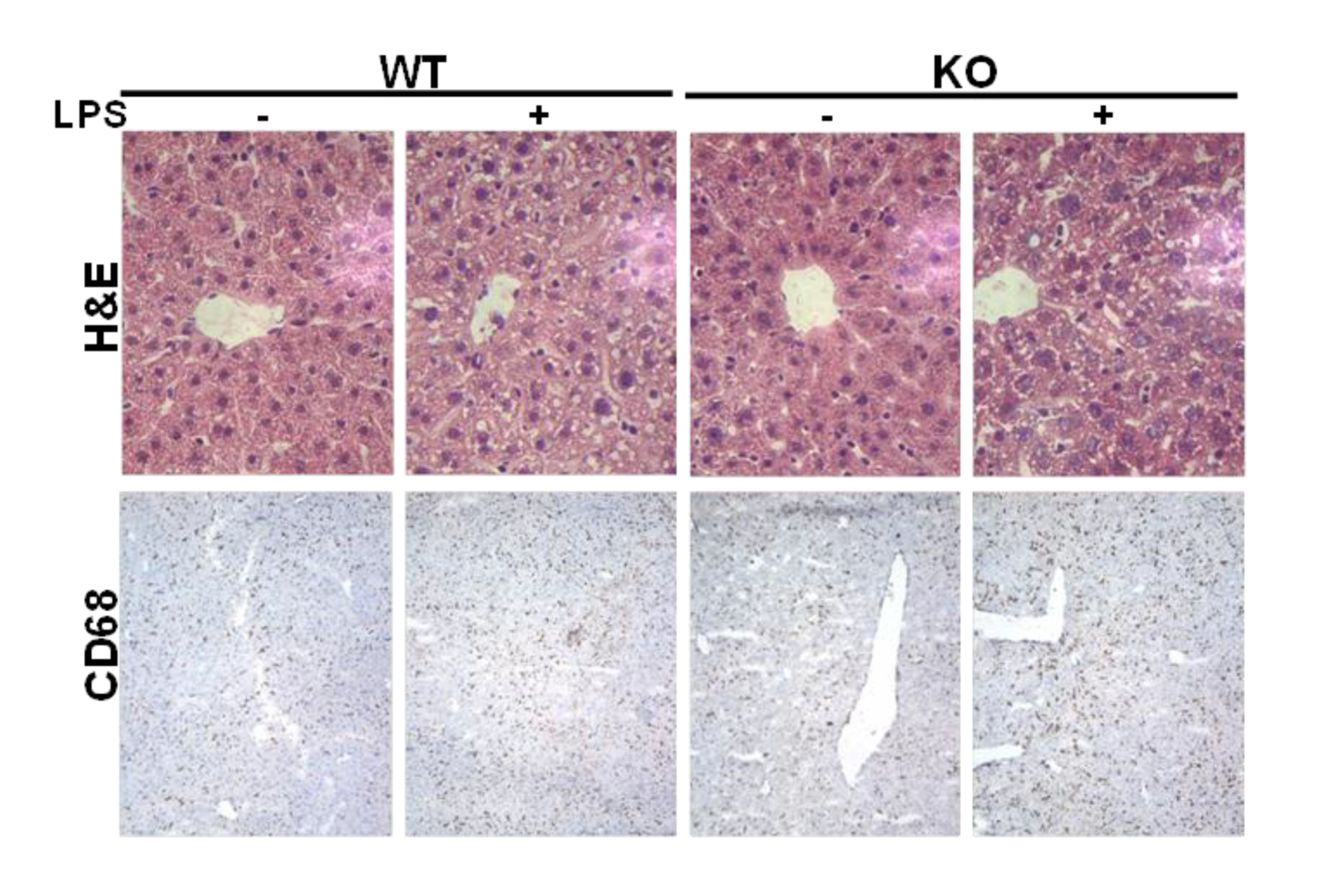

Supplement: Figure S1 — The Zip14 null mutation did not produce liver damage as there was no detectable change in CD68, a marker of macrophage infiltration. (TIF) [file pone.0048679.s001.tif]

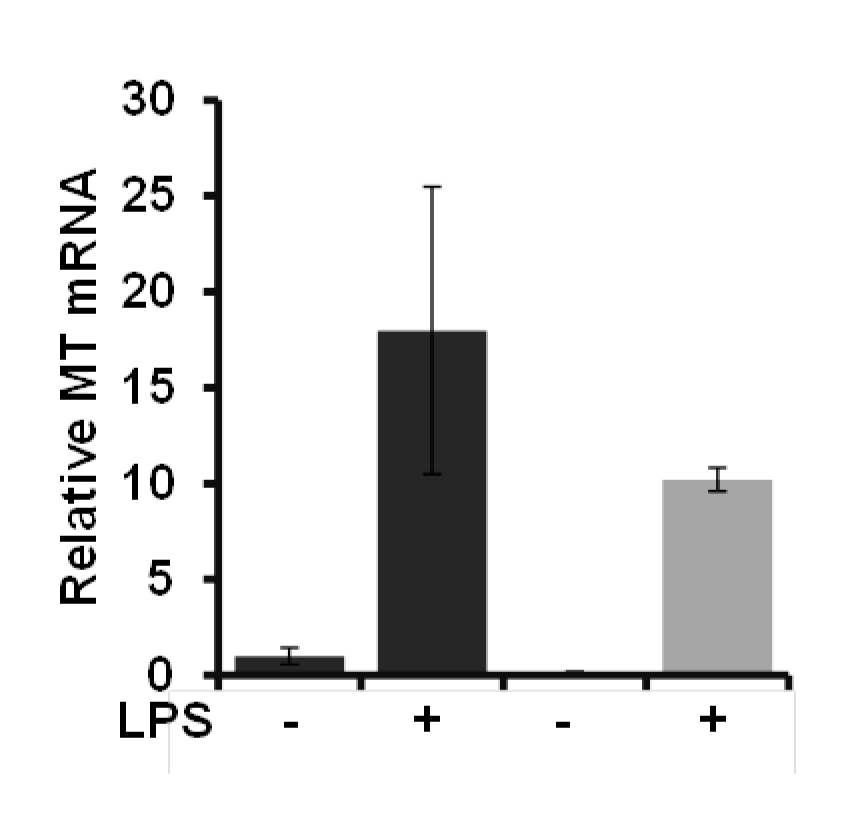

Supplement: Figure S2 — Comparison of MT mRNA WT and Zip14 KO mice 18 hr after LPS, as measured by qPCR. (TIF) [file pone.0048679.s002.tif]

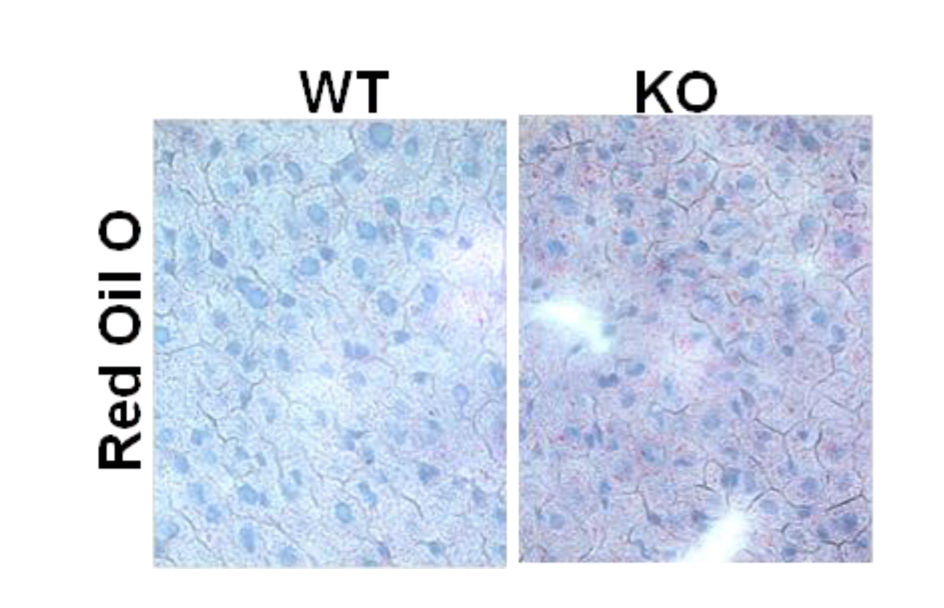

Supplement: Figure S3 — The increase in hepatic lipid content was shown by increased Red Oil O staining in the null mice. (TIF) [file pone.0048679.s003.tif]

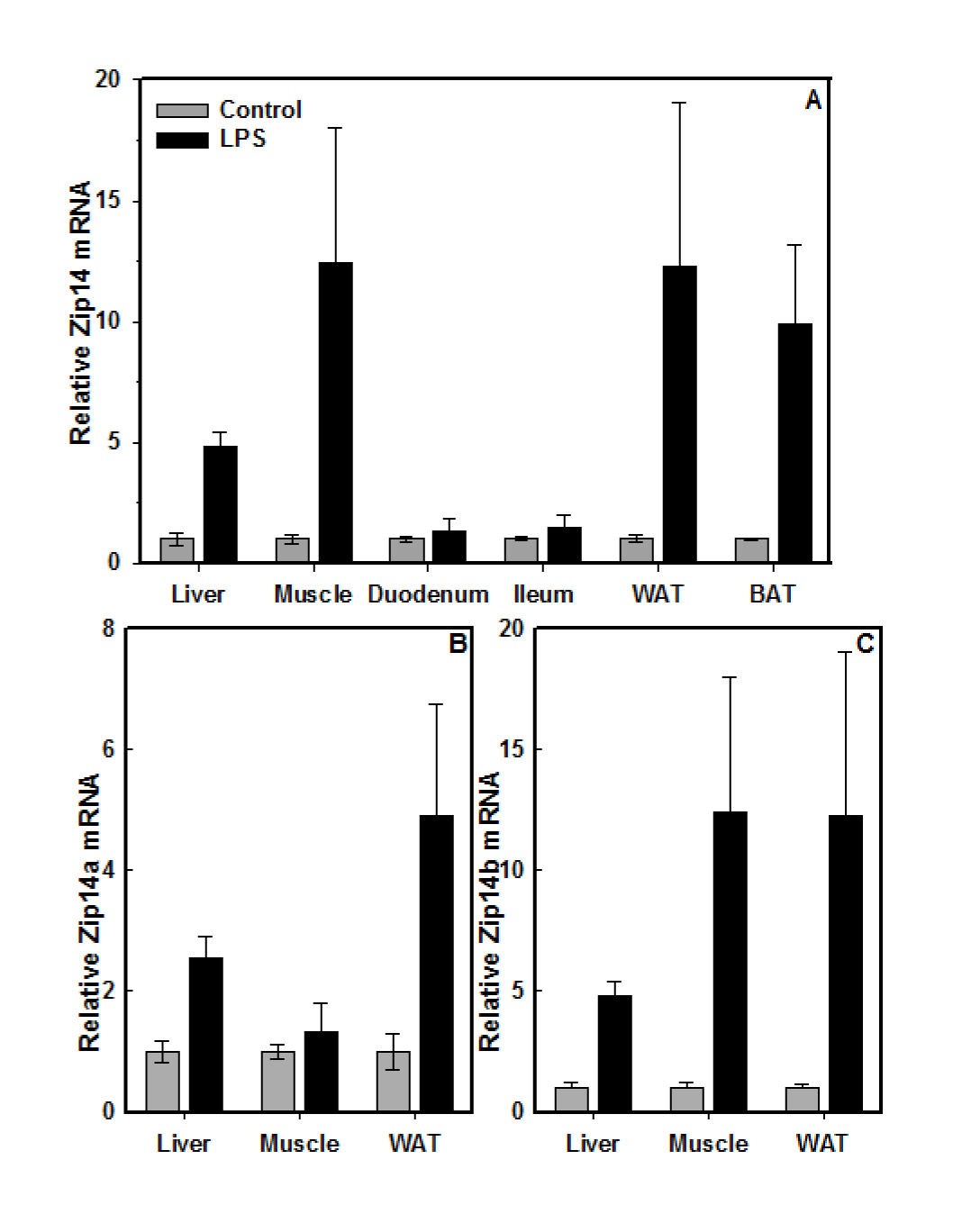

Supplement: Figure S4 — Zip14 mRNA abundance in CD-1 male mice and was measured in tissues after LPS. Zip14a and Zip14b transcripts were measured in liver, WAT and muscle. (TIF) [file pone.0048679.s004.tif]
